# Supplementary material for: Rhamnolipids bio-production and miscellaneous applications towards green technologies: a literature review
Source: PeerJ. 2025 Feb 21;13:e18981. doi: 10.7717/peerj.18981 (PMC12005046; doi:10.7717/peerj.18981)
Supplement: Supplemental Information 1 — Abdel-Mawgoud, Lépine & Déziel (2010). [file peerj-13-18981-s001.docx]

|  | **Symbol** | **M. Form.** | **MW** | **R_1_** | **n_1_** | **n_2_** | **R_2_** |
| --- | --- | --- | --- | --- | --- | --- | --- |
|  | **Rha-C_14:2_** | **C20H34O7** | **386.48** | **H** | **7(--4H)** | **--** | **H** |
|  | **Rha-C_10_-C_10:1_** | **C26H46O9** | **502.64** | **H** | **3** | **3(--2H)** | **H** |
|  | **Rha-C_12_-C_10_** | **C28H52O9** | **532.71** | **H** | **5** | **3** | **H** |
|  | **Rha-C_10_-C ^a^** | **C30H56O9** | **560.76** | **H** | **3** | **7** | **H** |
|  | **Rha-C_16_-C_16_** | **C38H72O9** | **672.97** | **H** | **9** | **9** | **H** |
|  | **Rha-Rha-C_12:1_** | **C24H42O11** | **506.58** | **H** | **5(--2H)** | **--** | **H** |
|  | **Rha-C_12:1_-C_10_** | **C28H50O9** | **530.69** | **H** | **5(--2H)** | **3** | **H** |
|  | **Rha-C_8_-C_12_** | **C26H48O9** | **504.65** | **H** | **1** | **5** | **H** |
|  | **Rha-C_10_-C_10_-CH_3_** | **C27H50O9** | **518.68** | **H** | **3** | **3** | **CH_3_** |
|  | **Rha-Rha-C_12_-C_12:1_** | **C36H64O13** | **704.89** | **H** | **5** | **5(--2H)** | **H** |
|  | **Rha-Rha-C_14_-C_12_** | **C38H70O13** | **734.95** | **H** | **7** | **5** | **H** |
|  | **Rha-Rha-C_14_-C_14_** | **C40H74O13** | **763.00** | **H** | **7** | **7** | **H** |
|  | **Rha-Rha-C_12_-C_12_** | **C36H66O13** | **706.90** | **H** | **5** | **5** | **H** |
|  | **Rha-Rha-C_10_-C_14:1_** | **C36H64O13** | **704.89** | **H** | **3** | **7(--2H)** | **H** |
|  | **Rha-Rha-C_12:1_-C_10_** | **C34H60O13** | **676.83** | **H** | **5(--2H)** | **3** | **H** |
|  | **Rha-C_12_-C_8_** | **C26H48O9** | **504.65** | **H** | **5** | **1** | **H** |
|  | **Rha-C_10_-C_12:1_** | **C28H50O9** | **530.69** | **H** | **3** | **5(--2H)** | **H** |
|  | **Rha-C_10_-C ^a^** | **C30H54O9** | **558.74** | **H** | **3** | **7(--2H)** | **H** |
|  | **Rha-C_10_-C_12_** | **C28H52O9** | **532.71** | **H** | **3** | **5** | **H** |
|  | **Rha-C_12_-C_10_** | **C28H52O9** | **532.71** | **H** | **5** | **3** | **H** |
|  | **Rha-C_12_-C ^a^12:1** | **C30H54O9** | **558.74** | **H** | **5** | **5(--2H)** | **H** |
|  |  |  |  |  |  |  |  |
|  | **Rha-Rha-C_8_-C_8_** | **C28H50O13** | **594.69** | **H** | **1** | **1** | **H** |
|  | **Rha-Rha-C_10_-C_10:1_** | **C32H56O13** | **648.78** | **H** | **3** | **3(--2H)** | **H** |
|  | **Rha-C_10_-C_10_-CH_3_** | **C27H50O9** | **518.68** | **H** | **3** | **3** | **CH_3_** |
|  | **Decenoyl-Rha-C_10_-C_10_** | **C36H64O10** | **656.89** | **b** | **3** | **3** | **H** |
|  | **Rha-Rha-C_14_-C_16_** | **C42H78O13** | **791.06** | **H** | **7** | **9** | **H** |
|  | **Rha-Rha-C_8_-C_12:1_** | **C32H56O13** | **648.78** | **H** | **1** | **5(--2H)** | **H** |
|  | **Rha-Rha-C_12_** | **C24H44O11** | **508.60** | **H** | **5** | **--** | **H** |
|  | **Rha-Rha-C_10_-C_12_** | **C34H62O13** | **678.84** | **H** | **3** | **5** | **H** |
|  | **Rha-Rha-C_12:1_-C_8_** | **C32H56O13** | **648.78** | **H** | **5(--2H)** | **1** | **H** |
|  | **Rha-Rha-C_8_-C_10_** | **C30H54O13** | **622.74** | **H** | **1** | **3** | **H** |
|  | **Rha-Rha-C_8_** | **C20H36O11** | **452.49** | **H** | **1** | **--** | **H** |
|  | **Rha-Rha-C_10_-C_8_** | **C30H54O13** | **622.74** | **H** | **3** | **1** | **H** |
|  | **Rha-Rha-C_10_-C_12:1_** | **C34H60O13** | **676.83** | **H** | **3** | **5(--2H)** | **H** |
|  | **Rha-Rha-C_12_-C_14_** | **C38H70O13** | **734.95** | **H** | **5** | **7** | **H** |
|  | **Rha-C_8:2_** | **C14H22O7** | **302.32** | **H** | **1(--4H)** | **--** | **H** |
