# Supplementary material for: Rhamnolipids bio-production and miscellaneous applications towards green technologies: a literature review
Source: PeerJ. 2025 Feb 21;13:e18981. doi: 10.7717/peerj.18981 (PMC12005046; doi:10.7717/peerj.18981)
Supplement: Supplemental Information 2 — Eslami, Hajfarajollah & Bazsefidpa (2020). NM, not mentioned. [file peerj-13-18981-s002.docx]

| Strain | Source | Substrate and growth condition | Extraction procedure |
| --- | --- | --- | --- |
| *P. aeruginosa* USM AR2 | A local crude oil sample Source | - Culture medium: 0.6% (w/v)  yeast extract, 0.05% (w/v)  MgSO47H2O, 0.05% (v/v) Tween80, and 30 mL diesel oil/pH 5.0/27 ◦C | - Centrifugation |
| *P. aeruginosa* (ATCC  10145) | American Type Culture  Collection | - Culture medium: 50% olive oil mill wastewater or whey  - 100 or 200 rpm  - 30 or 37 ◦C/PH 7.0/96 h | - Centrifugation, acidification, and  extraction using ethyl acetate |
| *P. aeruginosa* LBI |  | Mineral slat medium  - 30 ◦C | - Centrifugation and absorption |
| *P. aeruginosa* S6 | Oil-containing wastewater | Nutrient medium  - 165 rpm/30 ◦C/pH 7.5/48 h | Centrifugation and acidification |
| *P. aeruginosa* DR1 | Rice rhizosphere | Mineral slat medium with  diﬀerent concentrations/30 ◦C  - Harvesting 24 h till 120 h | Centrifugation, acidification, and extraction with chloroform–methanol |
| *P. aeruginosa* P20 | Institute of Medical  Research, Malaysia | - Mineral slat medium  - Carbon source: 1% (v/v) crude oil  - 150 rpm/40 ◦C/7 d | - Centrifugation and solvent  extraction |
| *P. aeruginosa* 57RP | A hydrocarbon-  contaminated soil | - Iron-limited mineral salts  medium supplemented with 2% (w/v) mannitol  - 150 rpm/30 ◦C/pH 6.7/ 359 h | - Centrifugation and filtration |
| *P. aeruginosa* ATCC  9027 | American Type Culture  Collection | PPGAS medium  - 250 rpm  - 37 ◦C | Centrifugation, acidification, and extraction with chloroform–ethanol |
| *P. aeruginosa* strain-PP2 | Soil  Contaminated with oil | Crude whey/150 rpm/ 30 ◦C  - pH 7.0/96h | - Centrifugation |
| *P. aeruginosa* PA1 | NM ^a^ | Culture medium (g L—1): NaNO3 1.0, KH2PO4 3.0, K2HPO4 7.0,  MgSO47H2O 0.2, 0.5% yeast  extract, peptone 0.5%, and 3% | - Centrifugation, using reverse osmosis process, and purification |
| *P. aeruginosa* BYK-2  KCTC 18012P | The southern sea of Korea | Basal salts medium  - Fish oil and urea as the carbonand nitrogen source  - 180 rpm/25 ◦C/40 h | Centrifugation |
| *P. aeruginosa* SP4 | Petroleum-contaminated  soil (Thailand) | - Mineral medium + palm oil  - 200 rpm  - 37 ◦C/22 h | Centrifugation |
| *P. aeruginosa* CPCL  (GQ241355) | A petroleum contaminated  site located in Chennai | - Mineral medium  - pH 7.0 0.2 | - Acidification, extraction  And concentration |
| *P. fluorescens* PMMD3 | The biofilm formed on  metal coupons  India | Minimal salt medium  - Paraﬃn as carbon source  - 35 ◦C | - Acidification and extraction using  equal volume of chloroform and ethanol (2 : 1) mixture |
| *P. aeruginosa* PAO1 | Source | Substrate and growth condition  - 37 ◦C/6 d (batch) and 10 d | Extraction procedure |
| *P. aeruginosa* DN1 | Petroleum contaminated  soil | - BPLM supplemented with palm oil and sodium nitrate as the nitrogen/ 7 d | - Centrifugation, and extraction using chloroform and methanol ethyl acetate |
| *P. aeruginosa* 6k11 | Soil with  crude-oil (Talara, Peru) | Mineral salt medium  - 140 rpm  - 37 ◦C  - pH 6.8/250 h | Centrifugation, acidification, and extraction using ethanol and  chloroform |
| *P. aeruginosa* PAO1 | NM | - BM2 minimal medium  - 170 rpm  - 37 ◦C/6 and 24 h | Solvent extraction  - Freezing and using subsequent phase separation |
| *P. aeruginosa* PAO1 | M. Foglino, Marseille,  France | PPGAS medium  - 37 ◦C  - pH 7.2 | Identifying and quantifying  rhamnolipids using LC-MS |
| *P. cepacia* CCT669 | The culture collection of  the Andr´e Tosello | Mineral medium  - 200 rpm/27 ◦C/pH 7 | - Centrifugation, acidification |
| *P. aeruginosa* 57RP | Hydrocarbon  contaminated soil | - Iron-limited mineral salts  medium (MSM) 2% (w/v) mannitol/200 rpm | - Centrifugation and filtration  - Adding an internal standard (hydroxyhexadecanoic acid) |
| *P. aeruginosa* ICP70 | Oily sludge | - Culture medium (per dm—3 of drinking water): glycerol, 30.5 cm3; MgSO4, 0.1 g; K2HPO4, 7 g; KH2PO4, 3 g; (NH4)2SO4  - 140 rpm/305 K/pH 6.5–7.0 | Thioglycolic acid method |
| *P. aeruginosa* ATCC  9027 | Source  American Type Culture  Collection | Substrate and growth condition  - Mineral salts medium  - 200 rpm/37 ◦C/pH 7.0/96 h | - Extraction procedure  - Centrifugation  - Concentrating  - Chromatography |
