# Supplementary material for: Rhamnolipids bio-production and miscellaneous applications towards green technologies: a literature review
Source: PeerJ. 2025 Feb 21;13:e18981. doi: 10.7717/peerj.18981 (PMC12005046; doi:10.7717/peerj.18981)
Supplement: Supplemental Information 3 — Nitschke & Costa (2007), Rahman & Gakpe (2008). [file peerj-13-18981-s003.docx]

| Type of surfactant | Microorganism |
| --- | --- |
| Glycolipids | Alcanivorax borkumensis, Arthrobacter sp., Corynebacterium sp., R. erythropolis, Serratia marcescens, Tsukamurella sp. |
| Lichenysin A, Lichenysin B | Bacillus licheniformis |
| Ornithine, lysine peptides | *Thiobacillus thiooxidans*, *Streptomyces sioyaensis*, *Gluconobacter cerinus* |
| Fatty acids (corynomycolic acids, spiculisporic acids, etc.) | *Capnocytophaga* sp., *Penicillium spiculisporum*, *Corynebacterium lepus*, *Arthrobacter paraffineus*, *Talaramyces trachyspermus*, *Nocardia erythropolis* |
| Alasan | *Acinetobacter radioresistens* |
| Surfactin | *Bacillus subtilis*, *Bacillus pumilus* |
| Diglycosyl diglycerides | *Lactobacillus fermentii* |
| Lipopolysaccharides | *Acinetobacter calcoaceticus* (RAG1), *Pseudomonas* sp., *Candida lipolytica* |
| Arthrofactin | *Arthrobacter* sp. |
| Lichenysin A, Lichenysin B | *Bacillus licheniformis* |
| Surfactin | *Bacillus subtilis*, *Bacillus pumilus* |
| Cellobiose lipids | *Ustilago maydis* |
| Trehalose lipids | *Arthrobacter paraffineus*, *Corynebacterium* sp., *Mycobacterium* sp.,  *Rhodococus erythropolis*,  *Nocardia* sp. |
| Lipopolysaccharides | *Acinetobacter calcoaceticus* (RAG1), *Pseudomonas* sp., *Candida lipolytica* |
| Phospholipids | *Acinetobacter* sp. |
| Viscosin | *Pseudomonas fluorescens* |
| Sulfonylipids | *T. thiooxidans*, *Corynebacterium alkanolyticum* |
| Sophorose lipids | *Candida apicola*, *Candida bombicola*, *Candida lipolytica*, *Candida bogoriensis* |
| Diglycosyl diglycerides | *Lactobacillus fermentii* |
